# EANM guideline on the validation of analytical methods for radiopharmaceuticals

**DOI:** 10.1186/s41181-019-0086-z

**Published:** 2020-02-12

**Authors:** Nic Gillings, Sergio Todde, Martin Behe, Clemens Decristoforo, Philip Elsinga, Valentina Ferrari, Olaug Hjelstuen, Petra Kolenc Peitl, Jacek Koziorowski, Peter Laverman, Thomas L. Mindt, Meltem Ocak, Marianne Patt

**Affiliations:** 1 Department of Clinical Physiology, Nuclear Medicine and PET, Copenhagen University Hospital Rigshospitalet, Blegdamsvej 9, DK-2100 Copenhagen, Denmark; 2 Tecnomed Foundation, University of Milano – Bicocca, Milan, Italy; 3 Center for Radiopharmaceutical Sciences, Paul Scherrer Institute, Villigen, Switzerland; 4 Department of Nuclear Medicine, Medical University Innsbruck, Innsbruck, Austria; 5 Department of Nuclear Medicine and Molecular Imaging, University Medical Center Groningen, Groningen, The Netherlands; 6 MSD Animal Health, Walton, UK; 7 Institute for Energy Technology (IFE), Kjeller, Norway; 8 University Medical Centre Ljubljana, Ljubljana, Slovenia; 9 Independant consultant, Linköbing, Sweden; 10 Radboud University Medical Center Nijmegen, Nijmegen, The Netherlands; 11 Ludwig Boltzmann Institute Applied Diagnostics, Vienna, Austria; 12 Department of Pharmaceutical Technology, Istanbul University, Istanbul, Turkey; 13 Department for Nuclear Medicine, University Hospital Leipzig, Leipzig, Germany

**Keywords:** Radiopharmaceuticals, Validation, Radioanalytical methods

## Abstract

**Background:**

To fulfil good manufacturing requirements, analytical methods for the analysis of pharmaceuticals for human and vetinary use must be validated. The International Conference on Harmonization of Technical Requirements for Registration of Pharmaceuticals for Human Use (ICH) has published guidance documents on the requirements for such validation activities and these have been adopted by the European Medicines Agency, The U.S. Food and Drug Administration (FDA) and other regulatory bodies. These guidance documents do not, however, fully address all the specific tests required for the analysis of radiopharmaceuticals. This guideline attempts to rectify this shortcoming, by recommending approaches to validate such methods.

**Results:**

Recommedations for the validation of analytical methods which are specific for radiopharmaceutials are presented in this guideline, along with two practical examples.

**Conclusions:**

In order to comply with good manufacturing practice, analytical methods for radiopharmaceuticals for human use should be validated.

## Preamble

The European Association of Nuclear Medicine (EANM) is a professional non-profit medical association that facilitates communication worldwide among individuals pursuing clinical and research excellence in nuclear medicine. The EANM was founded in 1985.

This guideline has been written by members of the EANM Radiopharmacy Committee and is intended to assist professionals involved in the preparation and quality control of radiopharmaceuticals to determine when and how analytical methods should be validated.

## Background

Radiopharmaceutical preparations or radiopharmaceuticals (RPs) are medicinal products which, when ready for use, contain one or more radionuclides included for a medical purpose. The radioactive compounds in RPs may contain simple salts (e.g. [^131^I]sodium iodide), metal complexes (e.g., [^99m^Tc]technetium exametazime), small organic molecules (e.g. [^18^F]fluorodeoxyglucose) or large molecules (e.g. ^125^I-labelled human serum albumin) as the active pharmaceutical ingredient. The principal radioactive ingredient may be characterised and quantified on the basis of the chemistry of the molecule and the physical properties of the radionuclide. As for any other pharmaceutical, their quality needs to be controlled before administration to patients, to ensure that their characteristics (i.e. identity, strength, and purity) are suitable for the intended purpose. However, for quality control of radiopharmaceuticals two specific aspects which differ from conventional pharmaceuticals must be taken into account:

- The strength of a radiopharmaceutical is defined by its radioactivity content (in MBq), or radioactivity concentration (MBq/ml), and it follows the decay law; thus, the strength of a radiopharmaceutical decreases with time. Radionuclides used in the field of molecular imaging and therapy may have half-lives in the range of seconds to hundreds of days.
- Whilst analytical techniques used to determine the content of non-radioactive components (e.g. precursors, cold ligands, non-radioactive impurities, residual solvents, etc.) of radiopharmaceutical preparations are generally the same as those used for conventional pharmaceuticals, radioactivity determination requires specific techniques, which make use of dedicated instrumentation capable of specifically detecting, discriminating and quantifying the radioactivity in the sample.

Before their use in routine quality control procedures, analytical methods must be validated. Validation is intended to ensure that the methods are suitable for their intended purpose. This involves testing a number of parameters as defined in the guidance document released by the International Conference on Harmonization of Technical Requirements for Registration of Pharmaceuticals for Human Use (ICH), which has been adopted by the European Medicines Agency (CPMP/ICH/381/95 2014) and is thus applicable in the Member States of the European Union (EU). The requirements for validation according to ICH can be seen in Table 1.

**Table 1:** Characteristics to be validated following ICH Q2(R1)

	Type of analytical procedure			
	Identification	Testing for impurities		Assay
		Quantitative	Limit	Dissolution Measurement only Content / potency
Characteristic				
Accuracy	–	+	–	+
Precision				
Repeatability	–	+	–	+
Intermediate Precision	–	+^a^	–	+^a^
Specificity^b^	+	+	+	+
Detection Limit	–	-^c^	+	–
Quantification Limit	–	+	–	–
Linearity	–	+	–	+
Range	–	+	–	+

– signifies that this characteristic is not normally evaluated

+ signifies that this characteristic is normally evaluated

^a^in cases where reproducibility has been tested, intermediate precision is not needed

^b^lack of specificity of one analytical procedure could be compensated by other supporting analytical procedure(s)

^c^may be needed in some cases

## Discussion

The ICH guideline provides a definition for each of the mentioned validation characteristics and methodology, with practical hints on how to investigate specificity, linearity, etc.; thus, it represents a general and commonly accepted basis for the validation of analytical methods. However, in the ICH guidelines it is also stated that “*approaches other than those set forth in this guideline may be applicable and acceptable. It is the responsibility of the applicant to choose the validation procedure and protocol most suitable for their product”*, thus recognizing that the suggested methodology may not be fully applicable in special cases. Although they are not specifically mentioned in ICH text, radiopharmaceuticals are certainly a special case. With the aim to provide guidance on the validation of radioanalytical methods, Table 1 has been modified to address the specific tests required for radiopharmaceuticals (Table 2). Besides the ICH guidelines, there are numerous other publications describing the validation of analytical methods for conventional pharmaceuticals. Thus, this guidance document will focus mainly on the validation of radioanalytical methods, with conventional methods only touched upon in special cases.

**Table 2:** ICH table adapted to radiopharmaceuticals

Type of analytical procedure	Radioactivity Content (assay)	Radionuclide identity (approx. t½)	Radionuclide identity (spectrometry)	Radiochemical identity (HPLC/TLC)	Radionuclidic purity (limit test)	Radionuclidic purity (spectrometry after decay)	Radiochemical purity^a^ (HPLC/TLC)
Characteristics							
Accuracy	+	–	+	–	+	+	+
Precision (Repeatability)	+	+	–	–	–	(+)	(+)
Intermediate Precision	–	–	–	–	–	(+)	(+)
Specificity	+	+	+	+	+	+	+
Detection Limit	–	–	–	–	+	–	–
Quantification Limit	–	–	–	–	–	+	+
Linearity	+	+	–	–	–	+	+
Range	+	+	–	–	–	+	+

(+) not always possible (e.g. short half life, see text)

^a^radioenantiomeric purity measurements should be validated analogously

Before designing a protocol for validation of analytical methods, it is crucial to check which quality references apply and if these need to be supplemented by further quality requirements, as this may determine the extent of required validation. In the 38 member states of the Council of Europe, including all members of the European Union (EU), the European Pharmacopoeia (Ph. Eur.) is the single, official and legal point of reference for manufacturing and quality control standards for medicinal products, including radiopharmaceuticals. The European Pharmacopoeia contains general monographs and a very large number of individual monographs for substances for pharmaceutical use, written by groups of experts, based upon specifications of authorised and frequently used preparations in Ph. Eur. member states and adopted by the Ph. Eur. Commission. The contents and indications of these monographs are mandatory in all 38 member states. These texts apply to both industrial and academic/hospital-based manufacturers and for “*any medicinal product which is prepared in a pharmacy in accordance with the prescriptions of a pharmacopoeia and is intended to be supplied directly to the patients served by the pharmacy in question (commonly known as the officinal formula)*”.

In the *General Notices* of the Ph. Eur. it is stated that “*The test methods given in monographs and general chapters have been validated in accordance with accepted scientific practice and current recommendations on analytical validation. Unless otherwise stated in the monograph or general chapter, validation of the test methods by the analyst is not required”.* Thus, if a Ph. Eur. monograph exists, the quality control of a (radio)pharmaceutical is described in the monograph, i.e. which controls have to be performed, including the related method of analysis with experimental details (e.g. stationary phase, mobile phase, flow rate, wavelength in case of HPLC analysis with UV detector, etc.) and acceptance criteria. If the monograph is followed, the analytical methods described do not need to be validated but must be verified in each individual laboratory, to ensure that the method has been implemented properly (e.g. system suitability test, detector linearity and limit of quantification). This is especially important for radioanalytical methods, where precise radiodetection methods are not specified in the individual monographs. It may also be relevant to verify that the chosen formulation of the preparation does not interfere with the tests described in the monograph. Currently, over 70 monographs for radiopharmaceuticals are available, which include most of the more frequently used radiopharmaceuticals, such as technetium-99m preparations (prepared from licensed kits) and fludeoxyglucose (^18^F) injection ([^18^F]FDG). If a monograph for a radiopharmaceutical has not been published, or in case the monograph exists but for any reasons it is preferred to use a different method, the suitability of the individual analytical method(s) must be assessed and demonstrated by validation.

## Radioanalytical tests

### Radioactivity content (assay)

The radioactivity content of a radiopharmaceutical is often determined using a dose calibrator (ionisation chamber). Other methods such as the use of well counters (scintillation detectors) may also be suitable. Further guidance can be found in the European Pharmacopoeia (Ph. Eur. 2.2.66 2016). Validation for a given radionuclide will normally be assured by calibration and qualification of the measurement system, and method (product) specific validation is generally not a prerequisite.

#### Accuracy

Accuracy is assured by calibration using sources of radionuclides traceable to national or international standards (e.g. cæsium-137). A daily constancy check is normally performed as confirmation. Recalibration should be performed as and when necessary. Instrument manufacturers normally provide specifications for accuracy.

#### Precision (repeatability)

Repeatability may be easily measured by six repeated measurements using a representative amount of radioactivity. The half-life of the radionuclide used, the quantification limit and the linear range of the radiodetector should be taken into account when determining how many repetitions it is feasible to perform. Decay correction may be required for short-lived radionuclides, thus the measured radioactivity needs to be recalculated to t_0_ using the decay equation:

$$ {\mathrm{A}}_{\mathrm{o}}=\mathrm{A}/{\mathrm{e}}^{-\uplambda \mathrm{t}} $$

where:

*A*_*0*_ = activity at time 0

*A* = activity at time t

t = time delay in minutes

λ = decay constant (ln2/t_1/2_)

#### Specificity

Specificity must be confirmed unless tests have ruled out the presence of relevant quantities of impurities which may interfere with the measurement. Possible attenuation due to matrix effects and geometry effects should be considered.

#### Linearity/range

Linearity measurements are generally part of the instrument qualification and should be performed at least yearly for the radionuclides used and over the whole measurement range. The useable measurement range should be based on the manufacturer’s recommendations and the validation data.

### Radionuclide identity

Radionuclide identity is established by assessing the physical characteristics of the radionuclide’s emissions. The energy of the radiation can be determined using a gamma-ray or beta-particle spectrometer. Additionally, radionuclide identity can be confirmed by approximate half-life measurements using a dose calibrator or spectrometer (gamma or beta). As can be seen in Table 2, different validation parameters are required for each test. Gamma-ray spectrometry is of limited usefulness for identification when the sample may include different radionuclides emitting gamma-rays of the same energy. This is indeed the case for positron emitters, that always emit gamma photons at 511 keV (and a sum peak at 1022 keV), due to the annihilation of positrons with the surrounding electrons; here, an additional decay test may allow to discriminate between two different positron emitting radionuclides, thus contributing to the identification of the desired radionuclide. Half-life determinations may be performed using either a gamma-ray spectrometer or dose calibrator, although the latter is easier and quicker to use and usually available in any nuclear medicine department. Gamma-ray spectrometry is often applied to determine the presence of long-lived radionuclide contaminants after sufficient decay of the main radionuclide.

#### Half-life measurement

A preliminary knowledge of the potential contaminant radionuclides’ identities is very important, as their half-lives and amounts affect the experimental results. For instance, a longer-lived contaminant increases the overall sample decay time, and vice versa, and the effect is proportional to the difference between the half-lives of the desired radionuclide and contaminants. In case the latter are significantly shorter lived, compared with the intended radioisotope, it may be useful to wait before analysing the sample, to allow for sufficient decay of the impurity. The waiting time required for this should be defined during method validation.

##### Precision (repeatability)

Repeatability is normally determined during instrument qualification for a given radionuclide as described for radioactivity content.

##### Specificity

Half-life measurement should be specific for pure radionuclides. A suitable measurement time should be established during the validation procedure, depending on the half-life of the specific radionuclide. A minimum of three measurments should be used to determine the half-life.

##### Linearity/range

Linearity is determined within a specified range during instrument qualification for a given radionuclide as described for radioactivity content.

#### Spectrometry

##### Accuracy

As a gamma-ray spectrometry detector provides a response in terms of emission energies, it is of paramount importance that the detector itself is suitably calibrated for energy using traceable standards. This is performed during instrument qualification and is not specific to any radiopharmaceutical method.

##### Specificity

Ideally, specificity should be evaluated using a reference standard containing the intended radionuclide in combination with one or more of the expected contaminant radionuclides. However, this is often unpractical, due to unsuitable half-lives of the selected radionuclides or simply because they are not easily available from commercial sources. A useful alternative may be a calibrated, multi-nuclide or single-nuclide with multigamma-ray emission source, that may provide useful peaks in the intended working range (usually 0–2000 keV). As resolution is an indirect measure of specificity, results are strongly affected by the detector used, high purity germanium detectors having a much higher energy resolution than sodium iodide scintillation detectors (< 1% vs. > 10%). This factor should be accounted for in the conclusions related to the validation of the method. In practice, a series of measurements with the calibrated source(s) is performed, and the resolution factor (R_s_) is calculated considering the peak energies detected using the following equation:

$$ {R}_s=\frac{1.18\times \left(E{r}_b-E{r}_a\right)}{FWH{M}_a+ FWH{M}_b} $$

Where:

- Er_b_ = Energy of the peak for radionuclide « b »
- Er_a_ = Energy of the peak for radionuclide « a »
- FWHM_b_ = full width at half maximum peak height for radionuclide « b »
- FWHM_a_ = full width at half maximum peak height for radionuclide « a »

Acceptance criteria might be set to R_s_ > 1. In the case of sodium iodide detectors, Er_b_ and Er_a_ will be the centroid energies.

### Radiochemical identity

Identification takes advantage of the physical characteristics of the radionuclide and physicochemical characteristics of the radiopharmaceutical. As radiopharmaceuticals are present in tracer amounts, there is a large excess of the non-radioactive substance which is often detectable using standard physicochemical techniques such as measurement of UV absorbance. Chromatographic analyses such as HPLC, TLC or GC are generally required. The chromatographic comparison of the radioactive product peak (i.e. retention time or retardation factor) with its non-radioactive counterpart (reference standard) may be used as an identification test. In some cases, it may be appropriate to use two independent chromatographic methods to verify the identity of the radiopharmaceutical product. The ability of the method to determine the chemical identity of the radioactively labelled substance must be demonstrated. The retention time or retardation factor of the main radioactive product peak must correspond with the retention time of the non-radioactive reference standard. When using gas or liquid chromatography, the delay time between the in-line physicochemical (e.g. UV) and radioactivity detectors must be accounted for. Generally, retention times should correlate within ±5%. The reference standard can be analysed prior to the radioactive products and the two retention times compared. If retention times or retardation factors fluctuate, then the radioactive sample can be spiked with reference standard for a comparison in the same analysis.

#### Specificity

Method validation must demonstrate that the radioactive product is resolved from any potentially interfering radioactive impurities. Baseline separation is preferable (Rs > 1.5; when two peaks of equal size, and Gaussian-shaped; i.e. “perfect” peaks have less than 1% overlap).

### Radionuclidic purity

This is often divided into two tests:

- i) A limit test to determine the presence of short-lived radionuclides

This test is aimed at detecting impurities with half-lives comparable with that of the main radionuclide. For impurities with shorter half-lives, it is expected that their amount becomes negligible at the time the radiopharmaceutical is used. The test is often performed using a sodium iodide scintillation detector and additionally acts as a radionuclide identity test, where for example the characteristic 511 and 1022 keV peaks from positron emitters will be seen. Normally the main peak will hamper any attempts to detect small radionuclidic impurities and tests following decay of the main radionuclide are required (see below).

#### Accuracy

Accuracy relies on the energy calibration and is generally assured by instrument qualification as mentioned earlier.

#### Specificity

It should at least be verified that any potential impurities which may be present can be detected at the predefined limit. Measurement times and applied radioactivity should be established during the validation.

#### Detection limit

For known short-lived radionuclidic impurities, the measurement system and method should be optimised, and the detection limit should be determined. An estimation based on a signal to noise ratio of 3:1 as described in the European Pharmacopoeia (Ph. Eur. 2.2.46 2016) is appropriate.

- ii) A quantitative test (after decay) to determine the presence of longer-lived radionuclidic impurities.

The absence of long-lived radionuclides in radiopharmaceutical preparations must be ensured. For example, cyclotron produced zirconium-89 may contain yttrium-88 (t½ = 106 days) and fluorine-18 may contain tritium (t½ = 12.3 years) and various long-lived metal radionuclides leached from cyclotron target foils (e.g. manganese-52 and 54 and cobalt-56, 57 and 58). Radionuclides often have gamma emissions which are characteristic and unique, and by using suitable methods such as gamma-ray spectrometry, which is capable of detecting gamma-rays and their emission energies, it is possible to quantify any radionuclidic impurities. In the case of tritium, a beta detection system is essential as there are no gamma-ray emissions.

#### Accuracy

Accuracy may be evaluated using traceable single or multinuclide calibrated sources. It is important that the half-life of the calibration nuclide(s) is long enough to allow measurement with minimum effects due to decay. After a suitable number of acquisitions, accuracy is then determined by comparison of calculated activity of the calibration radionuclides with the activity quantified by the instrument.

The relative content of longer-lived radionuclide contaminants increases with time. Thus, it is important to quantify contaminants whose half-life is similar or longer compared with that of the main radionuclide. Samples should be allowed to decay for a suitable time, and decay time should be clearly defined. For instance, Ph. Eur. monographs for fluorine-18 labelled radiopharmaceuticals suggest retaining the sample at least 24 h to allow fluorine-18 to decay to a level that permits detection and quantification of the impurities. Most Ph. Eur. monographs specify a radionuclidic purity of > 99.9%. This should be demonstrated throughout the shelf-life of the radiopharmaceutical, which can be particularly challenging for short-lived radionuclides. For example, [^18^F]FDG often has a 12 h shelf-life. Thus, the radioactivity due to fluorine-18 decays by a factor of ca. 100 during this shelf-life and 0.1% of radionuclidic impurities must be quantifiable relative to the radioactivity of the product at the end of its shelf-life.

#### Precision (repeatability)

Measurement times and detector settings should be standardised to obtain a sufficient precision. Repeatability can be validated by six consecutive measurements of the same sample.

#### Intermediate precision

If considered appropriate, testing of the same sample on different days should be used to demonstrate intermediate precision.

#### Specificity

It should be verified that any potential impurities which may be present can be detected at the predefined limit using the measurement system. Measurement times should be established during the validation.

#### Detection limit

Using gamma-ray spectrometry, the limit of quantification may be suitably replaced by MDA (minimum detectable activity) values, which are determined by qualified software in the instrument every time a sample is measured. MDA is a parameter depending on several factors such as geometry, activity, background activity, counting time, etc. All these factors should be defined during validation, in order to obtain consistent results.

#### Linearity

Linearity will normally be demonstrated during instrument qualification.

#### Range

A suitable range should be defined, based on establishing measurement times, in order to ensure detection of potential radionuclidic impurities above the specified limit with defined sample radioactivities.

### Radiochemical purity

#### Radio-HPLC

Radiochemical purity (RCP) measurements establish the content of impurities labelled with the same radionuclide used to prepare a radiopharmaceutical, but with a different chemical form. Strictly speaking, determination of radiochemical purity is not truly quantitative, as it is typically calculated as the ratio between the peak area of the desired radiopharmaceutical and the overall area of all the detected peaks in the radiochromatogram (corrected for decay). The instrument used to determine radiochemical purity with HPLC (radio-HPLC) is the radiometric detector (radiodetector), which is normally an in-line detector connected in series with a UV or other physicochemical detector. The radiometric detector can be a Geiger-Müller probe, a scintillation detector or a PIN diode. The validation of the method relies on the fact that all the applied sample (radioactivity) is eluted from the HPLC column. This is known as recovery and, before any other validation parameters are considered, it is necessary to measure this.

##### Recovery

Certain radiochemical impurities commonly found in radiopharmaceuticals (e.g. [^18^F]fluoride, [^68^Ga]gallium ions, ^99m^Tc-pertechnetate) may be retained in the injection system, pre-column filters, tubing or the column material itself. There are several methods suitable for estimation of these effects, which must be considered when validating the method. For example:

- Comparison of the injected radioactivity with the eluted radioactivity – this can be achieved by collecting the eluent in fractions and measuring them (compared with the calculated or measured injected radioactivity).
- Performing a second analysis in which the sample bypasses the HPLC column and flows directly through the radiodetector (using switching valves) and comparing the peak areas from the two radiochromatograms, after appropriate correction for radioactive decay. This method will not account for retention of radioactivity in the injection system or tubing.
- By performing spike recovery experiments with samples spiked with known amounts of a radioactive impurity (e.g. [^18^F]fluoride). This method requires very careful sample preparation to be certain that the “true” radiochemical purities are accurate.

##### Accuracy

In some circumstances, it may be appropriate to determine accuracy of a method using non-radioactive reference materials. This relies on the availability and detectability of compounds which are detected as radioactive impurities. It can, however, be challenging to identify such impurities due to the low masses involved (may require highly sensitive MS detectors). In this situation accuracy may be determined during the validation of the method for the determination of the “cold” drug substance and chemical impurities. However, in this case, any characteristics specific to the radiodetector used will not be accounted for.

If the identity of radioactive impurities is known, and if they are available, then these can be utilised to determine accuracy, by preparing samples spiked with various amounts of impurities and comparing the expected RCP with the measured RCP. The results can be expressed as recovery in percentage terms. An example of the importance of this concept is the determination of radiochemical purity of fluorine-18 labelled RP’s. It is well known that free [^18^F]fluoride adheres to some extent to many HPLC columns and considerable tailing of the peak is often observed. This is dependent on the pH of the eluent and the column material (Ory et al. 2015). Thus, in order to validate a radiochemical purity method where [^18^F]fluoride is a specified impurity, a spike recovery analysis may be justified. If radio-HPLC alone is relied on for a radiochemical purity test, a radio-TLC method may also be useful to verify the accuracy of the method.

##### Precision (repeatability)

The repeatability of RCP determination should be determined by sequential analysis of at least 6 homogeneous samples of the radiopharmaceutical preparation with a radioactivity concentration close to that expected for routine analysis. However, the half-life of the radionuclide, the limit of quantification and the linear range of the radiodetector should be taken into account when determining how many repetitions it is feasible to perform. Peak areas obtained after integration need to be recalculated to T_0_ using the decay equation:

$$ {\mathrm{A}}_{\mathrm{o}}=\mathrm{A}/{\mathrm{e}}^{-\uplambda \mathrm{t}} $$

Where:

A_0_ = corrected peak area

A = measured peak area

t = time interval in minutes between the considered injection and the first one

t_1/2_ = half-life in minutes

λ = decay constant = ln2/t_1/2_

Peak areas, normalized for decay, may then be used to calculate the radiochemical purity and perform a statistical analysis. A specification for precision in absolute percentage terms should be defined for this test, e.g. RCP ± 0.5%. In cases where the radiopharmaceutical is of very high radiochemical purity, it may be necessary to spike samples with a known radioactive impurity in order to determine repeatability.

##### Intermediate precision

Since HPLC systems are generally automated, the results should be independent of the analyst and thus omission of this test may be justified unless manual injections are performed. The intermediate precision may however be assessed by having different analysts evaluate the chromatograms obtained during the repeatability tests. Manual integration of peaks can be subjective and may therefore affect the precision of the method.

##### Specificity

If the radiochemical species have non-radioactive (“cold”) counterparts available, the assessment performed during the validation of the method for the determination of the drug substance may be applicable to the method for radiochemical purity, as it may be assumed that retention times are comparable. However, any differences in peak width between “cold” and radioactive peaks should be considered. Where “cold” reference samples of radiochemical impurities are unavailable, retention times and the ability of the method to clearly separate them from the desired radiopharmaceutical should be assessed. A second HPLC method (different column or different mobile phase) may be considered to confirm that there are no radioactive impurities coeluting with the main peak. Radiopharmaceutical product samples should be spiked with radioactive reference standards, if available, to determine the resolution factor. Baseline resolution of all peaks is preferable (Rs > 1.5). As mentioned above, certain radioactive impurities such as [^18^F]fluoride may be challenging and a secondary complementary method may be necessary.

##### Quantification limit

Determination of the limit of quantification (LOQ) is important, as the expected activities of the radiolabelled impurities are usually low or very low, and experimental tests or calculations are aimed to determine the lowest activity at which they may be reliably quantified. In practice, considering that both the desired product and the impurities are labelled with the same radionuclide, LOQ may be experimentally determined using samples labelled with the desired radionuclide (for instance, the radiopharmaceutical itself), allowing them to decay, and performing the HPLC analysis. Whilst a determination of LOQ is achievable, this is always related to a specific substance (peak) and will not necessarily be appropriate for a given radioactive impurity. Thus, validation of LOQ should determine the amount of radioactivity (radioactivity concentration and injection volume) which must be applied in order to assure the quantification of small amounts of radioactive impurities. LOQ is determined in terms of absolute radioactivity or radioactivity concentration. These values must be related to the determination of RCP (in percentage terms) such that its uncertainty can be quantified. For example, it may be defined that the RCP of a radiopharmaceutical should be quantifiable to with 0.5%. In this case, the LOQ should be sufficient such that a 0.5% radioactive impurity can be reliably determined.

##### Linearity/range

For the determination of linearity, the same samples and results used for repeatability assessment may be used (depending on the half-life). Alternatively, sequential dilutions may be used. The calculated amount of radioactivity is plotted against the measured amounts and the linear fit should comply with R ≥ 0.99. As stated earlier, the linearity of radioactivity measurement systems is an integral part of an Operational Qualification (OQ) or Performance Qualification (PQ) and, if appropriate, these results may be sufficient. The method should be linear from LOQ to the highest expected radioactivity concentration for a sample.

For stability studies of short-lived radiopharmaceuticals, further validation may be required in order to assure accuracy of the results at the end of shelf-life.

##### Robustness

Robustness is normally assessed during the validation of the “cold” physicochemical HPLC validation (e.g. small changes in eluent composition and pH). For evaluation of radiochromatograms, there should be strict rules regarding peak integration and setting baselines. It may be appropriate to test the robustness of this by comparison of the integration of chromatograms by several different analysts (see intermediate precision).

#### Radio-TLC

Radio-TLC is a relatively simple technique but can be very useful if validated correctly. As compared with radio-HPLC it has the big advantage that all applied radioactivity is detected and there are no concerns with recovery. Consideration should however be given to the potential for volatile radioactive species based on knowledge of the synthetic pathway and degradation products. On the other hand, TLC is a less efficient chromatographic technique and resolution of similar compounds is often non-trivial if at all possible.

Validation of radio-TLC methods involves the same parameters as for radio-HPLC:

##### Accuracy

If known radioactive impurities are available, then accuracy can be verified by spiking real samples with known amounts of these impurities. Acceptance criteria similar to those proposed for radio-HPLC may be used.

##### Precision (repeatability)

As for radio-HPLC, repeated measurements of the same homogeneous sample should be performed. For radio-TLC, it is recommended to perform a minimum of 6 measurements. Sample application and the elution of TLC sheets can be somewhat variable and thus requirements for repeatability should reflect this. For short-lived or unstable radiopharmaceuticals, an assessment of repeatability may not be possible, and the qualification of the TLC scanner must be relied upon.

##### Intermediate precision

For radio-TLC, there can be differences in sample application and TLC plate development techniques. Therefore, measurement of a homogeneous sample analysed by different operators is a good way to assess this.

##### Specificity

As with radio-HPLC, specificity is reflected by the resolution of the product from known impurities. The specification is normally set to baseline separation, however, lower resolution may be justified in certain cases.

##### Quantification limit

The same considerations as for radio-HPLC should be applied.

##### Linearity/range

The same considerations as for radio-HPLC should be applied.

##### Robustness

This is an important parameter to be considered for radio-TLC. The treatment of TLC plates/sheets can strongly influence their performance. For example, using glass fiber sheets (iTLC sheets) it is important that they are properly stored, i.e. in a dry environment. The effects of deliberate small changes in methods should be evaluated. These might include, but not limited to, application volume, spot drying time, mobile phase composition, plate development time/distance etc.

## Other test methods for radiopharmaceuticals

Other general analytical methods may be of concern in the characterization of radiopharmaceuticals. The most important of these tests are considered below.

### Determination of organic solvents

Radiopharmaceuticals often contain small amounts of organic solvents as impurities. Furthermore, ethanol is often present in radiopharmaceutical formulations as a stabiliser. Gas chromatography is normally applied for these analyses, and these methods should be validated according to the general ICH guidelines. When present as an excipient, assessment of ethanol content should be considered an assay test. When present as a residual solvent its quantification is validated as a limit test as for other organic solvent impurities (e.g. acetonitrile, acetone, DMSO etc.).

### Determination of pH using a pH-meter

As the instrument and the analysis operating procedures are very simple and straightforward, analytical method validation tests may be used also for the qualification of the instrument itself (and vice versa). Precision and linearity may be easily determined using calibrated standard solutions, that embrace the intended pH working range (e.g. three standard solutions of pH 4, 7 and 10). Other ICH parameters do not apply. Determinations of the pH of radiopharmacuticals containing ethanol should be considered approximate. If pH strips are used, the accuracy of these for each radiopharmaceutical product should be validated using a calibrated pH-meter.

### Colour spot tests for Kryptofix^®^ or tetrabutylammonium ions

Kryptofix® 222 in specific radiopharmaceutical preparations may be analysed as described in their Ph. Eur. monographs, e.g. fludeoxyglucose (^18^F) injection (Ph. Eur. monograph no. 1325 2014) by means of a spot test or using other methods, such as GC (Ferrieri et al. 1993) analysis. In the former situation, validation may not be required, provided that Ph. Eur. methods are followed, whilst if a non-pharmacopoeial method is used, validation is required. For GC methods, the standard validation requirements for impurities should followed. A validated HPLC method is described in several Ph. Eur. monographs for analysis of tetrabutylammonium ions. There are also colour spot test methods published for the analysis of tetrabutylammonium ions (e.g. Kuntzsch et al. 2014), which should be validated before use.

## Revalidation

An analytical method should be re-validated in case of:

i) changes in the RP preparation process that may result in different impurities that have not been accounted for (e.g. when a purification method is changed, or a different precursor is used);
ii) changes in the composition of the finished product, for example higher radioactivity or change of excipients;
iii) significant modifications in analytical procedure; for example, the replacement of an existing HPLC column with a new one with a different stationary phase or significant changes to the eluent.

An objective method to evaluate the validation “status” of an analytical method is provided by system suitability tests (SST), which are usually performed prior to the experimental analyses. Should SST results suggest that the method is no longer suitable for the intended purpose, then a verification of maintenance, calibration and qualification status of instruments should be performed.

## Practical validation examples

Below are two real examples of validation of radioanalytical methods. It should be noted that the acceptance criteria for the various tests, which can be found in the validation results summary tables, are based on generally accepted values and are not intended to be hard and fast recommendations. These values may vary depending on the type of instrument and analysis.

### Validation of the determination of [^18^F]fluoride in [^18^F]fluoroethyl-L-tyrosine using radio-TLC

#### Aim

To demonstrate that the applied radio-TLC method is suitable for the intended purpose and complies with generally acceptable analytical method validation criteria and is thus fit for purpose.

During the course of any method validation, a certain amount of optimisation may be necessary in order to meet acceptance criteria.

#### References

ICH Topic Q2 (R1) Validation of Analytical Procedures: Text and Methodology (CPMP/ICH/381/95)

European Pharmacopoeia 9.5, fluoroethyl-L-tyrosine (^18^F) injection (07/2015:2466)

European Pharmacopoeia 9.5, 2.2.46 Chromatographic separation techniques (07/2016:20246)

#### Validation characteristics

For validation purposes, this analysis is considered as a test for radiochemical purity and the characteristics which should be considered for validation are listed in the table below.

**Table:** 

Type of analytical procedure	Radiochemical purity (HPLC/TLC/PC)
Characteristics	
Accuracy	+
Precision (Repeatability)	(+)
Intermediate Precision	(+)
Specificity	+
Detection Limit	–
Quantification Limit	+
Linearity	+
Range	+

+ normally evaluated

(+) - not always possible

**Table:** 

Type of analytical procedure	Radiochemical purity (HPLC/TLC/PC)
Characteristics	
Accuracy	+
Precision (Repeatability)	(+)
Intermediate Precision	(+)
Specificity	+
Detection Limit	–
Quantification Limit	+
Linearity	+
Range	+

+ normally evaluated

(+) - not always possible

## Methods and results

### Analytical method

A radio-TLC method for [^18^F]fluoroethyl-L-tyrosine (fluoroethyl-L-tyrosine (^18^F) injection, [^18^F]FET) is described in the European Pharmacopoiea (Ph. Eur.). This method is intended to quantify the content of the specified impurity, [^18^F]fluoride. The method validated here is very similar to the Ph. Eur. method with only minor modifications.

- TLC Plate: TLC Silica gel 60 on aluminium backing plate (Merck)
- Mobile phase: 70/30 (acetonitrile/25 mM sodium acetate buffer, pH 3.8)
- Application: 2 μl (10 mm from bottom of plate)
- Development: 80 mm from bottom of plate
- Drying: 1 min air drying at RT
- Detection: ScanRam TLC scanner with Laura 4^TM^ software (Lablogic) with a plastic scintillation detector, scan speed 0.2 mm/second, scan distance 100 mm.
- Retardation factors (R_F_): [^18^F]fluoride = 0; [^18^F]fluoroethyl-L-tyrosine = 0.7–0.8.

Raw data is automatically corrected for radioactive decay during the course of the TLC scan. Peaks are integrated and peak areas are derived using the software. An example of a chromatogram where a sample of [^18^F]FET has been spiked with 5% [^18^F]fluoride is shown below. As can be seen, the baseline is not completely flat. This is due to some slight streaking of the sample on the TLC plate and also some slight spillover of radioactivity. It is therefore important to define the integration procedures for both peaks (i.e. peak start and end distance).

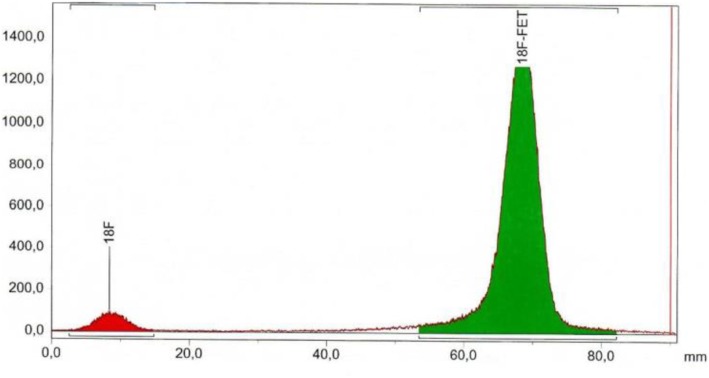



### Accuracy

Three samples of [^18^F]fluoroethyl-L-tyrosine were spiked with known amounts of [^18^F]fluoride and the exact [^18^F]fluoride content was calculated. Standard radio-TLC analysis was performed on each sample. Each sample was analysed twice and the measured [^18^F]fluoride content was averaged. The results and the expected (calculated or true) values were used to determine percentage recovery according to the equation:

$$ Recovery\ \left(\%\right)=\frac{Measured\  RCP}{Calculated\  RCP}.100 $$

**Table:** 

Accuracy					
Sample	Measured [^18^F]fluoride content (%)			True [^18^F]fluoride content (%)	Recovery (%)
	1	2	Average		
1	1.10	1.00	1.05	1.00	105.0
2	2.97	3.14	3.06	2.92	104.6
3	4.90	5.10	5.00	4.72	105.9

Careful preparation of samples is essential in order to ensure their accuracy. Adequate volumes of each component should be used to ensure accurate pipetting.

### Precision (repeatability)

A sample of [^18^F]FET spiked with [^18^F]fluoride (ca. 5%) was applied to six TLC plates which were developed and scanned. Standard deviation and variation coefficient (CV%) of the radiochemical purity were calculated.

**Table:** 

Repeatability	
Measurement no.	Measured [^18^F]fluoride (%)
1	5.2
2	5.3
3	5.3
4	5.1
5	5.4
6	5.3
Average (%)	5.27
Standard deviation	0.10
CV%	1.96

### Intermediate precision

The same sample of [^18^F]FET spiked with [^18^F]fluoride (ca. 5%) as used for the repeatability test was analysed by 2 different operators (samples applied, developed, scanned and evaluated) and the results compared.

**Table:** 

Intermediate Precision	
Analyst	Measured [^18^F]fluoride (%)
1	5.1
2	5.4
3	5.3
Average (%)	5.27
Standard deviation	0.15
CV%	2.90

### Specificity

For [^18^F]fluoroethyl-L-tyrosine, there are no known radioactive impurities besides [^18^F]fluoride, which may be present due to either inefficient purification or radiolysis of the product. Specificity is represented by the resolution of these 2 components and is calculated using the equation below (Ph. Eur. 2.2.46):

$$ Rs=\frac{1.18\alpha \left({R}_{F2-}{R}_{F1}\right)}{W_{h1}+{\mathrm{W}}_{\mathrm{h}2}} $$

*w*_*h*1_, *w*_*h*2_ = peak widths at half-height;

α = migration distance of the solvent front.

Results from the repeatability measurement were used to calculate the resolution

**Table:** 

Specificity	
Resolution of [^18^F]fluoride and [^18^F]FET	Rs = 5.7

### Limit of quantification

The quantification limit (LOQ) was measured by dilution of a sample and measurement until a signal to noise ratio (S/N) of ca. 10:1 was received. LOQ was determined for the specified impurity, [^18^F]fluoride only.

**Table:** 

Limit of Quantification	
LOQ	24 kBq/ml (S/*N* = 16: 1)

### Linearity

Five samples of [^18^F]fluoride were prepared by dilution to yield a suitable range of radioactivity concentrations. Samples were applied to TLC plates and these were scanned without development. All results were decay corrected to the start of the first measurement and a linear regression was applied to the results.

**Table:** 

Linearity			
Sample	Radioactivity concentration (MBq/ml)	Peak area (decay-corrected)	Correlation coefficient (R)
1	12.3	27,987	0.995
2	61.6	124,164	
3	123.2	209,463	
4	616.2	979,290	
5	1232.5	2,407,382	

### Range

The applicable radioactivity range for the samples to be analysed was calculated based on the product specifications. [^18^F]FET is produced in radioactivities up to 25 GBq in 23 ml. This equates to a radioactivity concentration of 1086 MBq/ml. The upper range of the linearity test was 1232 MBq/ml. The lower range limit can be derived from the LOQ based on a minimum detectable [^18^F]fluoride content of 0.5%. In order to meet this requirement, the applied radioactivity concentration should be 200 times higher than the LOQ, i.e. 24 × 200 = 4.8 MBq/ml. This equates to 110 MBq in 23 ml, which is below the minimum radioactivity specified for the product of 400 MBq. Thus, the validated analysis range is 4.8 - 1232 MBq/ml, which complies with the radioactivity specification for the product.

## Summary of results

Summary of the validation results can be seen below.

**Table:** 

Validation result summary				
Test Parameter	Acceptance Criteria		Result	
Accuracy	Recovery 90–110% ([^18^F]FET spiked with [^18^F]fluoride)		[^18^F]fluoride concentration	Recovery
			1%	105.0%
			3%	104.6%
			5%	105.9%
Repeatability	6 repetitions (5% [^18^F]fluoride)	RSD ≤5%	RSD = 1.96%	
Intermediate Precision	RSD ≤5% (3 analysts)		RSD = 2.90%	
Specificity	Resolution of > 2 between peaks		Resolution of[^18^F]fluoride and [^18^F]FET	Rs = 5.7
Limit of Quantification	S/N ratio ≥ 10		24 kBq/ml	
Linearity	12–1232 kBq/ml (5 concentrations in triplicate)	R > 0.99	R = 0.995	
Range	Reported value		4.8–1232 MBq/ml	

## Conclusions

The validation results have demonstrated that the method is acceptable with respect to the various test parameters and thus fit for purpose. It should be noted that the validated radioactivity concentration range should be taken into account when performing stability studies. For the [^18^F]FET product used in this validation example, a shelf life of 8 h is specified. Based on the analysis range derived from the validation, a minimum concentration of 4.8 MBq/ml should be available at the end of the shelf life. This equates to a concentration of 100 MBq/ml at the end of synthesis. Thus, for stability studies a minimum batch of 2.3 GBq in 23 ml should be available.

### Validation of a method for the determination of the radionuclidic purity after decay of [^18^F]fluorodeoxythymidine using gamma-ray spectrometry

#### Aim

To verify that the method used for determination of the radionuclidic purity after a minimum of 24 h decay of [^18^F]fluorodeoxythymidine ([^18^F]FLT) complies with the generally accepted validation criteria and is thus fit for purpose. A Ph. Eur. monograph exists for this product (Alovudine (^18^F) injection), where it is stated that the total radioactivity due to radionuclidic impurities, measured after a decay period of at least 24 h, should be not more that 0.1%.

#### References

- ICH Topic Q2 (R1) Validation of Analytical Procedures: Text and Methodology (CPMP/ICH/381/95)
- European Pharmacopoeia 9.5, alovudine (^**18**^F) injection (01/2014:2460)
- European Pharmacopoeia 9.5, 2.2.66 Detection and measurement of radioactivity

#### Validation characteristics

The characteristics to be considered during the validation of radionuclidic purity “after decay” are depicted in the following table:

**Table:** 

Type of analytical procedure	Radionuclidic purity (spectrometry after decay)
Characteristics	
Accuracy	+
Precision (Repeatability)	(+)
Intermediate Precision	(+)
Specificity	+
Detection Limit	–
Quantification Limit	+
Linearity	+
Range	+

+ normally evaluated

(+) - not always possible

## Methods and results

### Description of the analytical method

[^18^F]FLT (alovudine (^18^F) injection) is a radiopharmaceutical preparation labelled with the radionuclide F-18 (t_1/2_ = 109.77 min), obtained by the irradiation with 18 MeV protons of an enriched ^18^O-water target (O-18 > 95%) via the nuclear reaction ^18^O(p,n)^18^F. The radiopharmaceutical preparation may potentially contain contaminant radionuclides, formed by nuclear reactions between the incident proton beam and other isotopes contained in the target solution, as well as in the target holder material and window foil materials.

Radionuclidic purity, which is the ratio between activity of the desired radionuclide F-18, and total amount of radioactivity, may be determined using gamma-ray spectrometry, which is capable of identifying radionuclides exploiting their emitted energies, and whose response intensity (peak) is proportional to the amount of detected activity. Specification limits for radionuclidic purity of this preparation are defined in the European Pharmacopoeia monograph.

#### Measurement system

NaI Ortec, mod. Digibase 905–4, 3″ × 3″ crystal

Sample volume: 1 ml

Measurement time: 48 h after EOS

Measurement duration: 67 min

Calibration/validation sources (standards): multinuclide source with Eppendorf vial geometry, volume of 1.0 ml, containing the following radionuclides: Am-241, Cd-109, Co-57, Ce-139, Hg-203, Sn-113, Sr-85, Cs-137, Y-88 and Co-60.

The samples to be analysed were placed close to the detector surface. As the detector response is sensitive to various factors, such as geometry of the sample, volume, distance of the sample from the detector surface, etc., it is of paramount importance that all measurements are performed keeping all these parameters constant.

#### Potential impurities

The only potential radionuclidic impurity arising from the target materials itself (^18^O-water) is nitrogen-13. Due to its very short half-life, this will have fully decayed before the start of measurement. The target holder material is niobium, which is quite inert both from chemical and “nuclear” point of views, as there are no nuclear reactions with niobium capable of producing significant amounts of radionuclides at the applied proton energy. Thus, the major source of potential contaminants are the havar foils (target holder windows). Havar is an alloy made of seven different metals, and the most significant potential impurities are listed in the following table.

**Table:** 

Product	T_1/2_	Nuclear reaction	Threshold (MeV)
Co-55	17.5 h	^58^Ni(p,α)^55^Co	1.36
Co-56	77 d	^56^Fe(p,n)^56^Co	5.44
Co-57	272 d	^57^Fe(p,n)^57^Co	1.65
		^60^Ni(p,a)^57^Co	0.27
		^58^Ni(p, 2p)^57^Co	8.31
Co-58	71 d	^58^Fe(p,n)^58^Co	3.14
Ni-57	35.6 h	^58^Ni(p,pn)^57^Ni	12.43
Cr-51	27.7 d	^52^Cr(p,pn)^51^Cr	12.27
Mn-52	5.6 d	^52^Cr(p,n)^52^Mn	5.60
Tc-95	20 h	^95^Mo(p,n)^95^Tc	2.50
Tc-96	4.3 d	^96^Mo(p,n)^96^Tc	3.30
Re-181	19.9 h	^182^W(p, 2n)^181^Re	10.65
Mo-93 m	6.85 h	^93^Nb(p,n)^93m^Mo	3.60

The accuracy is the ratio between the experimental values and reference (or true) values. Thus, in case of gamma-ray spectrometry accuracy evaluation is intertwined with the calibration status of the detector in terms of efficiency. A couple of reference radionuclide sealed sources, of suitable energies and activities, have here been used to check the detector response.

For the evaluation of precision and intermediate precision, both [^18^F]FLT and reference sources may be used, through repeated acquisitions. When [^18^F]FLT is used, fast decrease of the activity with time should be accounted for by applying the decay equation.

Measurement of specificity would ideally require reference samples of the major expected contaminants, so as to discriminate between the various possible peaks. However, they are not all available in practice, and a multinuclide source covering a broad energy range has been used.

Quantification limit is the lowest activity that may be reliably quantified. To correctly evaluate this characteristic, reference standards of impurities should be available with appropriate radioactivity concentrations, but this is often not practical, and this parameter is thus verified using the major expected radionuclide as the reference.

### Accuracy

Accuracy was evaluated by 6 measurements of a reference source containing Co-60 (T_1/2_ = 5.27 y), with an expected activity, based on reference activity stated on the label, of 2546 Bq. Recovery was calculated as a percentage according to the equation:

$$ \mathrm{Recovery}:\frac{\exp value}{calculated\ source\ value}\times 100 $$

Where:

- Exp value is the activity experimentally determined by the gamma-ray spectrometer
- Calculated source value is the activity of the source stated on the label, corrected for decay if necessary

**Table:** 

Accuracy		
Expected activity: 2545.8 Bq		
Measurement no.	Measured activity (Bq)	Recovery (%)
1	2666.2	104.7
2	2722.9	106.9
3	2636.2	103.5
4	2714.8	106.6
5	2654.0	104.2
6	2654.3	104.2

### Precision (repeatability)

Precision was evaluated by measurement of repeatability using three different [^18^F]FLT samples which were each measured six times. Low activity samples were used, to match the activity range of potential impurities.

**Table:** 

Repeatability			
Test no. 1	Starting [^18^F]FLT activity at T_0_: 370 Bq		
Measurement no.	Measured activity (Bq)	Decay corrected activity (Bq)	
1 (T_0_)	370	370	
2 (T_1_)	362	372	
3 (T_2_)	353	371	
4 (T_3_)	341	368	
5 (T_4_)	328	363	
6 (T_5_)	326	370	
Average	369.4	Standard deviation	3.2
CV%	0.86%

**Table:** 

Repeatability			
Test no. 2	Starting [^18^F]FLT activity (at T_0_): 1320 Bq		
Measurement no.	Measured activity (Bq)	Decay corrected activity	
1 (T_0_)	1320	1320	
2 (T_1_)	1279	1311	
3 (T_2_)	1240	1305	
4 (T_3_)	1225	1322	
5 (T_4_)	1131	1307	
6 (T_5_)	1158	1314	
Average	1313	Standard deviation	6.8
CV%	0.52%

**Table:** 

Repeatability			
Test no. 3	Starting [^18^F]FLT activity at T_0_: 3021 Bq		
Measurement no.	Measured activity (Bq)	Decay corrected activity (Bq)	
1 (T_0_)	3021	3021	
2 (T_1_)	2933	3008	
3 (T_2_)	2853	3001	
4 (T_3_)	2793	3013	
5 (T_4_)	2710	2998	
6 (T_5_)	2659	3017	
Average	3010	Standard deviation	9.1
CV%	0.30%		

### Intermediate precision

Intermediate precision was assessed using a Co-60 calibrated source, by repeating the tests on three different days. The data obtained were evaluated with analysis of variance (Anova test). Intermediate precision is acceptable when the experimental value, F-_calc_ is less than F-_critical_ for the relevant number of degrees of freedom, at a confidence level of 0.05.

**Table:** 

Intermediate Precision			
Measurement no.	Radioactivity (Bq)		
	Day 1	Day 2	Day 3
1	895.0	858.0	870.0
2	867.0	868.0	858.0
3	867.0	879.0	868.0
4	868.0	866.0	869.0
5	871.0	880.0	879.0
6	875.0	865.0	857.0
Average	873.83	869.33	866.83
Variance	116.9667	73.4667	67.7667
Standard deviation	10.81511	8.57127	8.23205
CV%	1.24%	0.99%	0.95%

**Table:** 

Variance analysis			
Type of variation	Degrees of freedom (DF)	Average squares (AS)	F_calc_
Interaction between days	(n-1)	AS_B_	AS_B_/AS_Err_
Experimental error	n (r-1)	AS_Err_	
Total	nr-1		

Results: The experimental value, F_calc._ was 0.877, while F_critical_ was 3.68.

### Specificity

Specificity was evaluated using a calibrated, multi-nuclide reference source, which contains radionuclides whose gamma emissions cover a broad range of the energy spectrum, and whose difference in energies may resemble those of the expected contaminants previously listed in Table 1. Indeed, major gamma emission energies of the potential contaminant radionuclides are sufficiently separated from each other, and from the main 511 keV emission due to annihilation, such that they can be efficiently detected and quantitated using a NaI detector, whose resolution is typically in the order of 50 keV.

Peak resolutions were determined for “pairs” of gamma emissions (that is those which are closest in energy) using the equation described in the main guidance:

**Table:** 

Specificity			
Composition of the multi-nuclide source (in order of increasing energy emissions): Am-241, Cd-109, Co-57, Ce-139, Hg-203, Sn-113, Sr-85, Cs-137, Y-88, Co-60			
	Radionuclide	Energy (keV)	Resolution (Rs)
1	Am-241	59.5	Am-241/Cd-109: 1.82
2	Cd-109	88	Cd-109/Co-57: 1.92
3	Co-57	122	Sn-113/Co-57: 8.64
4	Sn-113	392	Cs-137/Sn-113: 4.47
5	Cs-137	662	Y-88(I)/Cs-137: 2.91
6	Y-88 (peak I)	898	Co-60(I)/Y-88(I): 2.94
7	Co-60 (peak I)	1173	Co-60(I)/Co-60(II): 1.44
8	Co-60 (peak II)	1837	Co-60(I)/Y-88(II): 3.62

### Linearity

As F-18 activity decreases rapidly with time, instead of preparing dilutions with different radioactivity concentrations, measurements of the same starting solution were performed, and peak areas corrected for decay. The radioactivity of the starting solution was quantified using a calibrated activimeter (dose calibrator).

**Table:** 

Linearity		
Starting activity of [^18^F]FLT: 16488 Bq		
Radioactivity (Bq)	Peak area (decay-corrected)	Correlation coefficient (R)
16,487.67	4,421,627	0.999
8629.16	2,314,149	
5644.31	1,513,678	
3712.19	995,526	
2241.00	654,623	
1615.31	433,190	
1054.80	282,874	
694.55	186,263	
460.02	123,367	
300.05	80,468	
194.77	71,629	
130.72	35,057	

### Quantification limit

The quantification limit was estimated using two reference sources of Co-58 (t_1/2_ = 70.86 d) and Co-57 (t_1/2_ = 271.74 d), respectively. Six measurements were performed with each of the two calibrated sources.

**Table:** 

Intermediate Presicion		
Mesurement no.	Co-57 radioactivity (Bq)	Co-57 radioactivity (Bq)
1	34.41	198.54
2	36.89	182.17
3	37.60	182.81
4	34.66	184.41
5	37.47	182.45
6	30.98	177.46
Average	35.34	184.64
Standard deviation	2.54	7.20
CV%	7.2	3.9

Based on these results, the limit of quantification is estimated to be 185 Bq. This will easily allow for detection of any significant radionuclidic impurities to ensure the product meets the specification for radionuclidic purity at the end of its shelf life (99.9%).

### Range

The applicable measurement range was defined based on the assessment of linearity and limit of quantification. The upper activity limit is determined by the intrinsic characteristics of detector response, which is known to be linear provided that deadtime does not exceed 5%. This experimentally corresponds to a value of ca. 16 kBq. The lower limit is set at 185 Bq based on the estimated limit of quantification.

## Summary of results

A summary of the validation results can be seen below.

**Table:** 

Validation result summary				
Test Parameter	Acceptance Criteria		Result	
Accuracy	Recovery 90–110%		Measurement	Recovery
			1	104.7%
			2	106.9%
			3	103.5%
			4	106.6%
			5	104.2%
			6	104.2%
Repeatability	6 repetitions using [^18^F]FLT, 3 different activities	%CV ≤5%	Radioactivity	%CV
			370 Bq	0.86%
			1320 Bq	0.52%
			3021 Bq	0.30%
Intermediate Precision	F_calc._ < F_critical_		F_calc._ = 0.877	F_critical_ = 3.68
Specificity	Resolution of > 1 between peaks		Peak Pair	Rs
			Am-241/Cd-109	1.82
			Cd-109/Co-57	1.92
			Sn-113/Co-57	8.64
			Cs-137/Sn-113	4.47
			Y-88(I)/Cs-137	2.91
			Co-60(I)/Y-88(I)	2.94
			Co-60(I)/Co-60(II)	1.44
			Co-60(I)/Y-88(II)	3.62
Limit of Quantification	Reported value		185 Bq	
Linearity	12 samples of F-18	R > 0.99	R = 0.999	
Range	Reported value		185 Bq - 16 kBq	

## Conclusions

The validation results have demonstrated that the method is acceptable with respect to the various test parameters and thus fit for purpose. The method is sensitive and accurate enough to enable determination of the radionuclidic purity of [^18^F]fluorodeoxythymidine with respect to long-lived radionuclides, in order to comply with the specifications for this preparation in the European Pharmacopoeia.

## Data Availability

Not applicable.

## Glossary

**Accuracy**: The accuracy of an analytical procedure expresses the closeness of agreement between the value which is accepted either as a conventional true value or an accepted reference value and the value found. This is sometimes termed trueness.
**Analytical procedure**: The analytical procedure refers to the way of performing the analysis. It should describe in detail the steps necessary to perform each analytical test. This may include but is not limited to: the sample, the reference standard and the preparation of reagents, use of the apparatus, generation of the calibration curve, use of the formulae for the calculation, etc.
**Detection limit**: The detection limit of an individual analytical procedure is the lowest amount of analyte in a sample which can be detected but not necessarily quantified as an exact value. This is normally expressed as limit of detection (LOD).
**Linearity**: The linearity of an analytical procedure is its ability (within a given range) to obtain test results which are directly proportional to the concentration (amount) of analyte in the sample.
**Precision**: The precision of an analytical procedure expresses the closeness of agreement (degree of scatter) between a series of measurements obtained from multiple sampling of the same homogeneous sample under the prescribed conditions. Precision may be considered at 3 levels: repeatability, intermediate precision and reproducibility. The precision of analytical procedure is usually expressed as the variance, standard deviation or coefficient of variation of a series of measurements.
**Repeatability**: The repeatability of an individual analytical procedure expresses the precision under the same operating conditions over a short time interval. Repeatability is also termed intra-assay precision.
**Intermediate precision**: The intermediate precision of an individual analytical procedure expresses within laboratory variations: different days, different analysts, different equipment, etc.
**Reproducibility**: The reproducibility of an individual analytical procedure expresses the precision between laboratories (collaborative studies, usually applied to standardisation of methodology).
**Quantification limit**: The quantification limit of an individual analytical procedure is the lowest amount of analyte in a sample which can be quantitatively determined with suitable precision and accuracy. The quantification limit is a parameter of quantitative assays for low levels of substances in sample matrices and is used particularly for the determination of impurities and/or degradation products. This is normally expressed as limit of quantification (LOQ).
**Radioactivity (assay, content or potency)**: (Assay, content or potency): Quantitative determination of radionuclide decay over time. For non-spectrometric methods of measurement of radioactivity like using ionization chambers, solid-state detectors (scintillation or semiconductors) and liquid scintillation, the detectors are in general unable to fully discriminate all radiations coming from different radionuclides. Thus, the reliability of these radioactivity measurements methods requires the assurance of the absence of interfering radionuclides (radionuclidic purity) or their relative contribution to the measurement results.
**Radiochemical purity**: The *radiochemical purity* of a radiopharmaceutical preparation represents that fraction of the radionuclide present in its stated chemical form.
**Radionuclidic purity**: The radionuclidic purity of a radiopharmaceutical preparation represents the proportion of the total radioactivity that is present as the required radionuclide.
**Range**: The range of an analytical procedure is the interval between the upper and lower concentration (amounts) of analyte in the sample (including these concentrations) for which it has been demonstrated that the analytical procedure has a suitable level of precision, accuracy and linearity.
**Recovery**: This term denotes the obtained result from an analytical procedure with respect to the true value of the sample under analysis and is often given as a percentage.
**Robustness**: The robustness of an analytical procedure is a measure of its capacity to remain unaffected by small but deliberate variations in method parameters and provides an indication of its reliability during normal usage.
**Specificity**: Specificity is the ability to assess unequivocally the analyte in the presence of components which may be expected to be present. Typically, these might include impurities, degradation products, matrixes, etc. Lack of specificity of an individual analytical procedure may be compensated by other supporting analytical procedure(s).: This definition has the following implications:: • Identification: to ensure the identity of an analyte.: • Purity tests: to ensure that all the analytical procedures performed allow an accurate statement of the content of impurities of an analyte, i.e. related substances test, heavy metals, residual solvents content, etc.: • Assay (content or potency): to provide an exact result which allows an accurate statement on the content or potency of the analyte in a sample.
